# Unraveling the Genetic Basis of Feed Efficiency in Cattle through Integrated DNA Methylation and CattleGTEx Analysis

**DOI:** 10.3390/genes14122121

**Published:** 2023-11-24

**Authors:** Zhenbin Hu, Clarissa Boschiero, Cong-Jun Li, Erin E. Connor, Ransom L. Baldwin, George E. Liu

**Affiliations:** 1 Animal Genomics and Improvement Laboratory, Beltsville Agricultural Research Center, Agricultural Research Service, United States Department of Agriculture, Beltsville, MD 20705, USA; 2 Department of Animal and Food Sciences, University of Delaware, Newark, DE 19716, USA

**Keywords:** feed efficiency, residual feed intake, DNA methylation, cattle

## Abstract

Feed costs can amount to 75 percent of the total overhead cost of raising cows for milk production. Meanwhile, the livestock industry is considered a significant contributor to global climate change due to the production of greenhouse gas emissions, such as methane. Indeed, the genetic basis of feed efficiency (FE) is of great interest to the animal research community. Here, we explore the epigenetic basis of FE to provide base knowledge for the development of genomic tools to improve FE in cattle. The methylation level of 37,554 CpG sites was quantified using a mammalian methylation array (HorvathMammalMethylChip40) for 48 Holstein cows with extreme residual feed intake (RFI). We identified 421 CpG sites related to 287 genes that were associated with RFI, several of which were previously associated with feeding or digestion issues. Activator of transcription and developmental regulation (*AUTS2*) is associated with digestive disorders in humans, while glycerol-3-phosphate dehydrogenase 2 (*GPD2*) encodes a protein on the inner mitochondrial membrane, which can regulate glucose utilization and fatty acid and triglyceride synthesis. The extensive expression and co-expression of these genes across diverse tissues indicate the complex regulation of FE in cattle. Our study provides insight into the epigenetic basis of RFI and gene targets to improve FE in dairy cattle.

## 1. Introduction

The dairy industry plays a vital role in the global food supply chain, providing essential nutrients and a wide range of dairy products [1]. Feeding cost is a significant cost to the cattle industry, which can be up to 75% of the total cost of cattle production [2]. Meanwhile, the livestock industry has a role in global climate change, contributing equal greenhouse gas emissions, especially when emissions due to transport and crop harvest are considered [3,4]. Increasing feed efficiency (FE) can reduce costs to the cattle industry and lower the environmental impact of dairy production by reducing enteric methane (CH_4_) emissions [2].

Residual feed intake (RFI) is an estimate of FE and is defined as the difference between the actual and expected feed intake based on animal size and production level, including body weight gain and energy-corrected milk yield. After accounting for the environment, time, management, and diet, the RFI from different studies can be compared directly to estimate the genetic components of FE variation. A negative RFI means high feed efficiency, while a positive RFI indicates low feed efficiency. In Holstein cattle, the RFI is estimated to have sufficient variation and heritability (*h*^2^), ranging from 0.17 to 0.27 [5,6,7] to enable genetic selection for increased FE. On average, the most efficient cows produce the same amount of milk as the least efficient cows, but with feed consumption of 1.6 to 3.7 kg less dry matter intake per day [8,9,10]. 

Researchers have investigated factors besides nutritional management and feed additives contributing to the variation in FE of Holstein and Jersey cows at the levels of DNA (genome), RNA (differentially expressed genes via RNA-seq), protein (enzymes), metabolism, and the gut microbiome [11,12,13,14,15,16]. A collaboration of nutritionists and geneticists funded by grants from the USDA National Institute of Food and Agriculture and Foundation for Food and Agriculture as well as the Center for Dairy Cattle Breeding has created a 6000-cow dataset to enable genomic selection for improved FE and reduced enteric CH_4_ emissions. The results of this work are being translated to the U.S. dairy industry through a trait called Feed Saved (released in December 2020), which has been incorporated into the Holstein Association’s Total Performance Index and the U.S. Net Merit Index. However, the improvement of FE based on genomic selection is challenging due to its low-to-intermediate heritability in cattle [17].

Additionally, many studies have focused specifically on the liver [18,19,20,21], mammary gland [22,23,24,25,26], and gastrointestinal tract of ruminants [27,28,29] to identify factors that contribute to animal-to-animal variation in the RFI. For example, RNA-seq and metabolomic results from liver and blood suggest that pathways in fatty acid and amino acid oxidation, immune response, and maintenance contribute to differing nutrient use between high- and low-FE animals [30,31].

Epigenetics refers to heritable changes in gene expression or cellular phenotypes that occur without alterations to the DNA sequence. It includes DNA methylation, histone codes, ncRNA, and chromatin states. Methylation of DNA is an epigenetic modification that involves adding a methyl group to the DNA molecule, which can influence gene expression and, consequently, various phenotypic traits. As DNA methylation is involved in many physiological aspects, such as fertility, reproduction, epigenetic inheritance, health, and disease, it has significant implications for understanding complex traits, phenotypic variations, environmental and nutritional influences, adaptation, and evolution. However, studies have not yet explored the epigenetics of FE in livestock, especially regarding DNA methylation.

Multiple methods can be used to study DNA methylation patterns, including methylation-specific PCR, methylated DNA immunoprecipitation, and bisulfite sequencing. However, they are rarely used in large-scale epigenetic studies due to limited coverage and throughput or high cost. In contrast, DNA methylation arrays, like the Infinium Methylation BeadChip (Illumina, San Diego, CA, USA), are commonly used for the high-throughput profiling of DNA methylation at CpG sites in human genomes. These arrays provide genome-wide methylation data and can simultaneously analyze thousands of CpG sites in multiple samples. They offer a balance between coverage, throughput, and cost, making them widely used in large-scale epigenetic studies. Recently, an Infinium microarray platform (GPL28271, HorvathMammalMethylChip40, Illumina Inc., San Diego, CA, USA) was designed [32] and used to generate DNA methylation data from numerous mammals [33,34,35,36,37,38,39,40,41]. 

This study aims to identify the epigenomic basis of FE, estimated as the RFI, using a 36 K mammalian methylation array [32] and CattleGTEx data to explore the genetic basis of FE. We identified pathways and candidate genes associated with FE in dairy cattle, providing essential epigenomic substrates for genome-enabled improvement.

## 2. Materials and Methods

### 2.1. Cows and Determination of RFI

The research dairy herd at the Beltsville Agricultural Research Center (BARC), Agricultural Research Service, U.S. Department of Agriculture (ARS-USDA) is representative of the U.S. Holstein population and serves as a suitable model for this work. At BARC, Holstein cows are routinely enrolled in FE studies using GrowSafe feeders (Vytelle, LLC; Lenexa, KS, USA), as described previously [42]. The cows were phenotyped for RFI and enteric CH_4_ emissions using an automated detection system (GreenFeed, C-Lock, Rapid City, SD, USA) in their first and second lactations in cohort groups for 42 days between 50 and 200 days of their milk production cycle. During these studies, the dry matter intake and milk yield were measured daily; milk samples were collected at all milkings 2 days/week for milk components analysis; and body weight was measured on 3 consecutive days in the first, middle, and last weeks of the study. Feed component and total mixed ration feed samples were collected weekly for subsequent dry matter and chemical composition analysis. 

**Figure 1:**
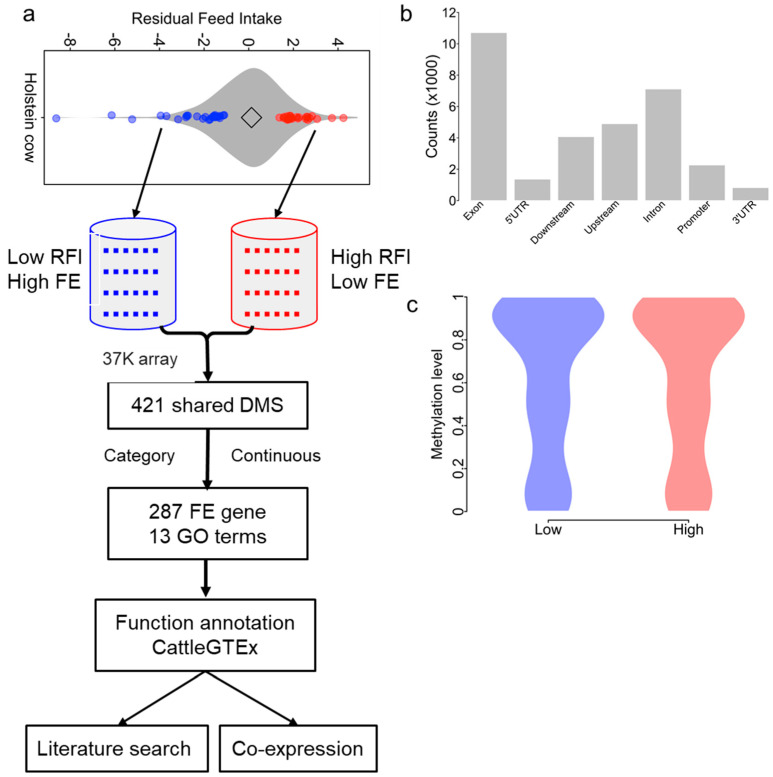
Flowchart of the experiment. (**a**) The experimental design. The violin plot shows the distribution of the **r**esidual **f**eeding **i**ntake (RFI) of 448 individuals. The blue dots represent the 24 selected low RFI (high **f**eed **e**fficiency: FE) individuals. The red dots are the 24 selected high RFI (low FE) individuals. The CpG and RFI association was identified using the dmpfinder tool by treating RFI separately as categorical or continuous. Their differential methylation CpG sites (DMS) were further explored using CattleGTEx data and a literature search. (**b**) CpG site distribution along gene features. UTR: untranslated region. (**c**) The methylation level distribution of CpG sites and comparison between low and high RFI groups. The methylation was normalized as *β*.

Calculation of the RFI is by statistical determination of the deviation of the actual dry matter intake of a cow from the expected intake, based on the average of the cohort (cows managed the same and fed the same diet at the same time) after adjusting for major energy sinks. The residual error term is the RFI [43]. From the RFI distribution histogram of our herd (Figure 1a), we selected 24 animals with low RFI values and 24 animals with high RFI values and individually measured each of their DNA methylation levels. A negative RFI value generally indicates better feed efficiency, while a positive RFI value indicates poorer feed efficiency.

### 2.2. DNA Methylation and Data Processing

Genomic DNA was extracted from the blood using the DNeasy Blood & Tissue Kits (Qiagen, Germantown, MD, USA). The DNA concentration was measured using Qubit (Thermo Fisher Scientific, Waltham, MA, USA). DNA samples were sent to the Clock Foundation at the Harbor-UCLA Medical Center (Torrance, CA, USA) for DNA methylation analysis. DNA methylation was measured using a custom Illumina methylation array (‘HorvathMammalMethyl40’), which was conserved across mammalian species [32]; the probes were aligned to the cattle genome assembly (ARS-UCD1.3). Raw .idat files were processed using the *minfi* (1.48.0) and *SeSAMe* (1.20.0) packages [44,45], and normalized methylation levels (*β* value) were generated at the probe on a scale between 0 (not methylated) and 1 (fully methylated) using the *SeSAMe* (1.20.0) package [45]. 

### 2.3. Associated CpG Site for RFI Identification

We categorized 48 cows into 2 groups based on their RFI values (Figure 1a). The differences in methylation levels between the groups for each CpG site were identified using the dmpFinder function with type = “categorical” in the *minfi* (1.48.0) package in R. The RFI was also treated as a quantitative measurement. The association between the RFI and methylation level was identified using the regression method *dmpFinder* function with type = “continuous” in the *minfi* package. In both approaches, a *q*-value ≤ 0.05 was used as a threshold to determine a CpG site associated with the RFI.

### 2.4. GO Enrichment Analysis

Gene ontology (GO) enrichment was conducted using the *TopGO* package (2.52.0) with algorithm = “weight01” and statistic = “fisher” [46]. Significant terms were identified at a *p*-value ≤ 0.01.

### 2.5. Co-Expression Using Expression Data from CattleGTEx

We downloaded the gene expression (transcripts per million-TPM) data from CattleGTEx [47] to build a co-expression network by using the CpG sites associated with the genes. The co-expression network was built using the weighted gene co-expression network analysis (WGCNA) package (v1.71) [48]. The soft threshold was identified using the *pickSoftThreshold* function of the *WGCNA* package. The module was identified using the *cutreeDynamic* function with deepSplit = 4 of the *WGCNA*. Modules were merged based on a similarity of <0.25. The hub gene for each module was identified using the *chooseTopHubInEachModule* function in the WGCNA. The co-expression network was visualized for TOM > 0.06 using the *GGNET* package (0.1.0).

## 3. Results

### 3.1. Experimental Design

Feed efficiency is a complex trait essential for reducing the cost of production in the dairy industry. Greater FE would benefit the environment by decreasing the release of greenhouse gases, particularly CH_4_. In this study, we designed a pool-like approach to dissect the epigenomic basis of FE (Figure 1a), estimated using the RFI, which is negatively correlated with FE. We selected 48 extreme FE individuals based on their RFI (24 cows with low RFI estimates and 24 cows with high RFI estimates) from the RFI data of 448 Holstein cows (Figure 1a). To identify the RFI-associated CpG sites, the methylation level of 48 individuals was quantified using a custom Illumina methylation array (HorvathMammalMethylChip40) [32], and two statistical approaches were used. First, the difference in CpG sites between the two groups was tested using an *F*-test by treating the two groups as categories (Figure 1a). Second, the regression between the methylation level and RFI was calculated. Then, the overlapped CpG sites between the two approaches were identified as final RFI-associated CpG sites (RFI-CpG) for further analysis.

### 3.2. Methylation Data Generation and Summarization

The methylation levels for the 48 selected cattle were successfully quantified for 37,554 CpG sites using DNA from blood (Figure 1b and Table S1). Those CpG sites were related to 5095 genes. A total of 28.52% and 18.94% of CpG sites were located in exons and introns, respectively (Figure 1b and Table S1). The distribution of the methylation levels of those CpG sites clearly showed three peaks: low, intermediate, and high (Figure 1c). The comparison between the low and high RFI groups did not show differences in the methylation level, on average (Figure 1c).

### 3.3. Identification of Differential Methylation CpG Sites between High and Low RFI Groups

**Figure 2:**
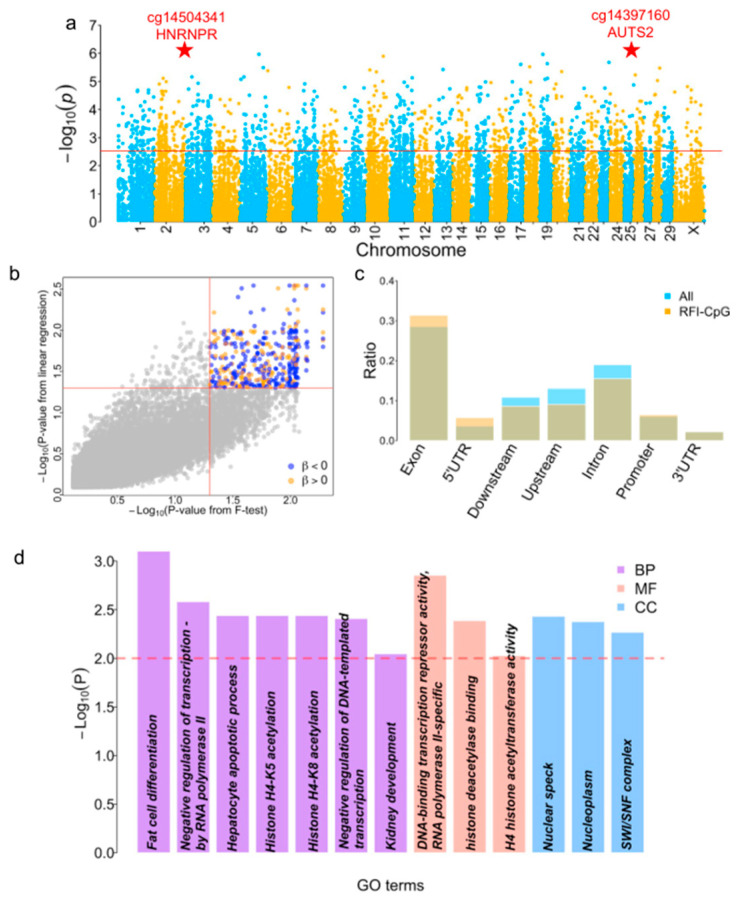
Identification of RFI−CpG associations. (**a**) Manhattan plot showing the RFI-CpG associations from a pool-based approach. The red line is the genome-wide threshold based on FDR < 0.05. The two top-associated CpG sites were annotated with red stars and related gene symbols. (**b**) Overlap between the F-test and linear regression. The yellow dot indicates the positive regression coefficient, and blue means the negative regression coefficient. (**c**) Distribution of the shared RFI-CpG using two approaches over all CpG sites related to gene features. (**d**) GO enrichment analysis of the FE-gene. GO enrichment analysis was performed using 5095 genes near CpG sites as the control. *p* < 0.01 was set as the threshold and is shown using a red dotted line. CC: Cellular component; BP: biological_process; MF: molecular function.

Although the above global results showed no differences in the methylation levels between the groups, the analysis could not reject specific sites that may have differed between the groups. To identify differential methylation CpG sites (DMS) for the RFI, we performed (1) an *F*-test by treating the RFI as categorical groups whereby the low RFI group was assigned when the RFI < 0 and the high RFI group was assigned when the RFI > 0; and (2) linear regression by treating the RFI as a continuous phenotype (Figure 1a). Both analyses were performed using the *minfi* package. We identified 1730 CpG sites related to 925 genes based on the false discovery rate (FDR) < 0.05 using an *F*-test (Figure 2a and Table S2). The linear regression identified 559 DMS involving 364 genes at FDR < 0.05 (Table S3). There were 421 shared DMS from the two approaches involving 287 genes (Figure 2b and Table S4). Then, we checked the direction concordance of the methylation and RFI in the two datasets, further narrowing the RFI-CpG sites to 156 sites positively correlated and 265 sites negatively correlated with the RFI (Figure 2b). The position distribution of 421 CpG sites related to genes shifted favorably for exons and 5′ UTR (Figure 2c). 

We performed GO term enrichment analyses for the 287 genes to identify the potential pathways associated with the RFI. Because this array was designed mainly based on conserved sequences among mammals [32], we only used the 5095 conserved genes targeted by CpG sites instead of all known genes to avoid bias in the GO enrichment. A total of 287 genes (FE genes) were enriched in 13 GO terms, including 7 biological processes, 3 molecular functions, and 3 cellular components (Figure 2d). The top enriched GO term was fat cell differentiation (GO:0045444), in which unspecialized cells acquire specialized features of an adipocyte to synthesize and store fat. Two transcription-related GO terms, GO:0000122 and GO:0045892, were enriched, suggesting the transcriptional difference between high- and low-RFI groups was caused by methylation. Histone H4 at the known acetylation sites K5 and K8 (GO:0043981 and GO:0043982) were also enriched. One GO term (GO:0001822) was enriched in kidney development. Therefore, the enrichment of different GO terms indicated multiple pathways involved in FE and the complex genetic regulation of FE in cattle.

### 3.4. Function Annotation of the FE Genes

**Figure 3:**
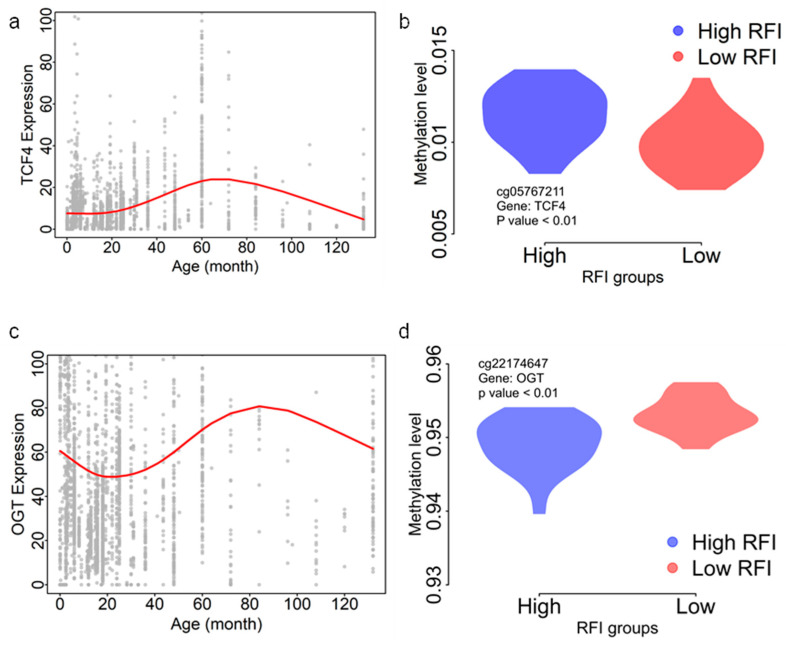
CpG gene expression throughout aging. (**a**) *TCF4* expression with cattle age increase. (**b**) Comparison between methylation levels of cg05767211 between low- and high-RFI groups. (**c**) *CGT* expression with cattle age increase. (**d**) Comparison between methylation levels of cg22174647 between low- and high-RFI groups. The red curves in (**a**,**c**) were modeled using the *smooth.spline* function in the R package for age and gene expression.

To query the expression patterns of the 287 FE genes, we used CattleGTEx data and filtered away the tissues with fewer than three repeats per tissue. These 287 genes were widely expressed across all remaining tissues (Figure S1). Previous studies showed a positive correlation between FE and age at the early life stage and a negative correlation at the late-life stage [49]. We hypothesized a similar relationship between FE gene expression and age. Thus, we analyzed the relationship between 287 FE genes and age using the CattleGTEx data [47], using 2863 samples with age data. The ages of the cattle were all normalized to months. When the RNA-seq for tissues was obtained from an embryo, the age was set to zero. The ages of these samples ranged from 0 to 132 months. Then, we analyzed the relationship between gene expression and age, with 48–60 months as the turning point of correlation. We identified 8 genes, including *ZDHHC17*, *ING4*, *TCF4*, *ZIC2*, *UBE4B*, *SOX21*, *OGT*, and *ADGRV1*, with opposing relationships at two different stages (Table S5). *TCF4,* a transcription factor, was negatively correlated (*ρ* = 0.06, *p* = 2.09 × 10^−3^) with age at the early stage and positively correlated (*ρ =* −0.16, *p =* 4.93 × 10^−4^) with age at the late stage (Figure 3a). There also were significant differences in methylation between the high- and low-RFI groups (Figure 3b). A previous study showed that *TCF4* influences glucose metabolism and is associated with fasting in mice [50]. *OGT* serves as a nutrition sensor by catalyzing the addition of *O-GlcNAc* to serine and threonine residues of hundreds of target proteins in a UDP–GlcNAc–dependent manner [51] and plays an important role in feeding behavior in mice [52]. It was negatively correlated (*ρ* = −0.07, *p* = 2.60 × 10^−4^) with age at the early stage and was positively correlated (*ρ* = 0.14, *p* = 2.12 × 10^−3^) with age at the late stage (Figure 3c). However, when we fit a smooth curve between age and gene expression, we observed a negative relationship at the early stage of 20 months (Figure 3c). The comparison between the low- and high-RFI groups shows significant differences (*p* < 0.01) in the methylation level (Figure 3d).

**Figure 4:**
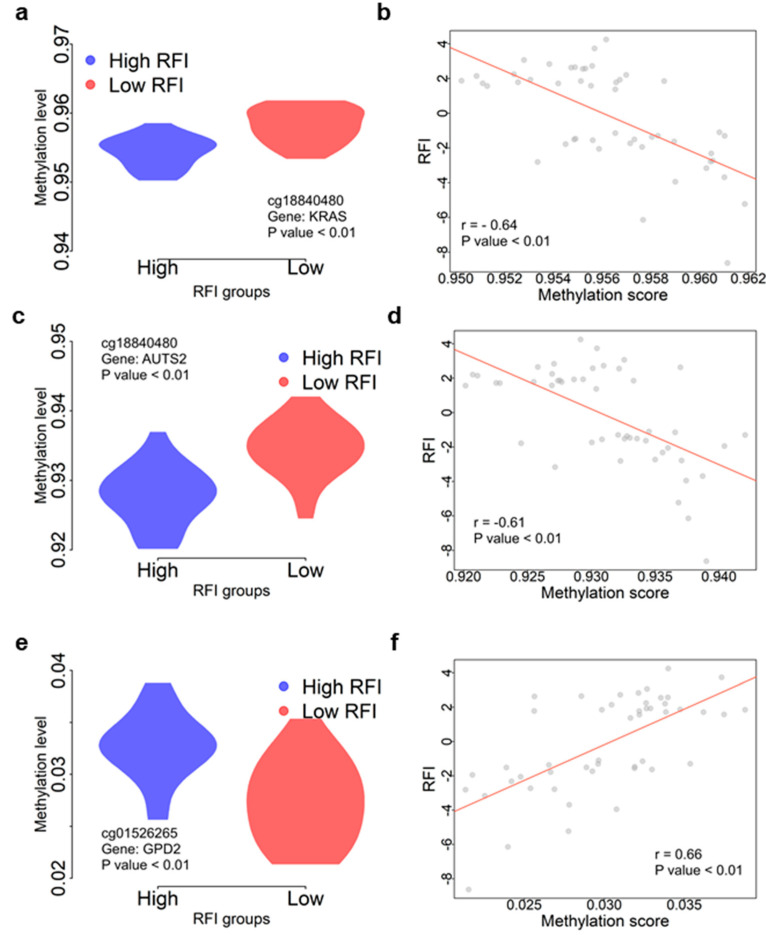
Functional annotation for FE genes. (**a**) The comparison between methylation levels of cg18840480 between low- and high-RFI groups. The corresponding gene (*KRAS*) of cg18840480 was annotated on the panel. (**b**) The correlation between methylation scores and RFI using 48 individuals for cg18840480. (**c**) The comparison between methylation level of cg14397160 between low- and high-RFI groups. The corresponding gene (*AUTS2*) of cg14397160 was annotated on the panel. (**d**) The correlation between methylation scores and RFI using 48 individuals for cg14397160. (**e**) The comparison between methylation levels of cg01526265 between low- and high-RFI groups. The corresponding gene (*GPD2*) of cg01526265 was annotated on the panel. (**f**) The correlation between methylation scores and RFI using 48 individuals for cg01526265. The red lines in (**b**,**d**,**f**) were regressions between two corresponding variables.

We also completed a literature search for other genes to find evidence supporting those genes associated with FE. CpG site cg18840480 was identified as the top CpG-RFI association in both approaches. It was localized in the exon of *KRAS* (Figure 4a). The methylation level of cg18840480 was negatively correlated (*r* = −0.64, *p* = 7.51 × 10^−7^) with the RFI (Figure 4b). *KRAS* was widely expressed across tissues (Figure S2). *KRAS* can interact with nutrients to contribute to human cell growth [53,54]. The CpG site cg14397160 was identified upstream of *AUTS2* using two approaches (*F*-test: *p* = 7.46 × 10^−7^ and *q* = 5.22 × 10^−3^; regression: *p* = 4.19 × 10^−6^ and *q* = 6.07 × 10^−3^) (Figure 4c,d). *AUTS2* is validated to function in human digestive problems [55,56]. *AUTS2* is also involved in neurodevelopment and is identified as a candidate gene for numerous neurological disorders [57]. CpG site cg01526265 was located upstream of *GPD2*, which regulates glucose utilization and fatty acid and triglyceride synthesis [58]. It was upregulated under nutrient deprivation and along with the accumulation of triacylglycerol or glycerol in *Chlamydomonas reinhardtii* [59]. *GPD2* also plays an important role in transferring nutrients into biomass in fruit flies [60]. It was positively correlated with RFI (*ρ* = 0.64, *p* = 2.09 × 10^−6^) (Figure 4e,f).

### 3.5. Co-Expression Network of FE

**Figure 5:**
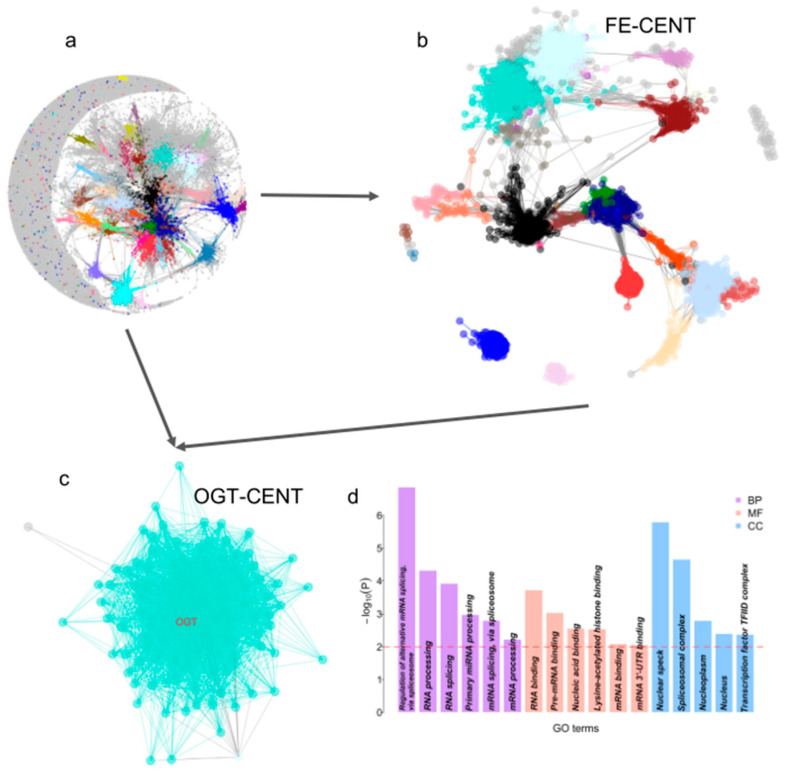
Co-expression network of FE genes. (**a**) Global co-expression networks using 27,538 genes across 5462 individual cattle. Different colors indicate various co-expression modules. The global co-expression network only included genes with correlation > 0.70. (**b**) Co-expression network of FE gene (FE-CENT). (**c**) Co-expression network of OGT (OGT-CENT). OGT is labeled as red color in the network. (**d**) GO enrichment for OGT co-expressed genes and different colors means three types of GO terms. BP, biological process, MF, molecular function, and CC, cellular components.

We used 287 FE genes and a co-expression network from CattleGTEx to explore the regulation network of RFI further [47]. Firstly, we built a co-expression network using CattleGTEx gene expression data and retained only the “good” genes by filtering with the *goodSamplesGenes* function in the WGCNA package [48]. Finally, we retrieved 27,538 genes across 5462 samples to build the co-expression network. We used the soft power of 16 to ensure a scale-free network and merged the highly similar modules using a similarity of 0.25. Finally, we identified 54 modules with module sizes ranging from 12,816 (grey) to 30 (yellow2) (Figure 5a and Table S6). The co-expression network displays a scale-free architecture with a clear modular topology (Figure 5a). The genes from each module were enriched in various GO terms, indicating that similar functional genes tended to co-express (Table S7). 

Then, we mapped 287 FE genes in the co-expression network and identified 4134 co-expressed genes for 68 of 287 FE genes based on the threshold (r > 0.75). Then, we built a sub-co-expression network (FE-CENT) (Figure 5b). Those genes were enriched in multiple GO terms with the top enriched GO terms related to the regulation of postsynaptic membrane potential (Figure S4).

Furthermore, we narrowed the co-expression network into a sub-co-expression network of OGT (OGT-CENT) (Figure 5c). OGT-CEN includes 121 genes, 119 of them from the turquoise module, one from the grey, and one from the light cyan module (Figure 5c). Gene enrichment analysis of the 121 genes showed they are enriched in 17 GO terms, which mainly involve RNA-related biological processes, such as the top enriched terms as a regulation of alternative mRNA splicing via spliceosome (Figure 5d). The results indicate that the regulation of RNA processing plays an important role in dairy cattle FE.

## 4. Discussion

Traditional genetic approaches have been applied to enhance FE by selectively breeding animals with superior performance [61]. However, the underlying molecular mechanisms of FE are intricate and involve both genetic and epigenetic factors. Epigenetic modifications, like DNA methylation, have pivotal roles in regulating gene expression patterns and influencing phenotypic variation [62]. For instance, researchers studying Holstein dairy cows have explored DNA methylation patterns to gain insights into milk production, fertility, disease resistance, and overall performance [63,64,65,66]. In our study, the CpG site for RFI was enriched in GO terms associated with transcription regulation (Figure 2d), suggesting that gene expression differences between high- and low-RFI groups may be mediated through DNA methylation. In a recent study, authors observed expression differences among cattle with different feed efficiencies using the RNA-seq approach [67,68].

Epigenome-wide association studies (EWAS) employing DNA methylation array data are a powerful tool for investigating the epigenetic basis of complex traits [69]. Through a rigorous selection process, we identified cattle with extreme feed efficiency phenotypes (high- and low-feed-efficient individuals), generated genome-wide DNA methylation data, and conducted an initial association between DNA methylation and RFI estimates. Our comprehensive analysis revealed numerous differentially methylated CpG sites distinguishing high- and low-feed-efficient individuals (Figure 2). These DMS were found near genes critical in multiple GO terms related to different biological activities (Figure 2d), suggesting the complex regulation network of FE traits. A previous study also observed methylation associated with FE or feed strategy [70,71]. Several genes highlighted in the present study (Figure 3 and Figure 4) were closely associated with nutrient digestion or validated in other species for digestive problems. Those genes provide essential targets for future molecular investigations of FE regulation in dairy cattle.

The co-expression of FE genes revealed candidate genes that may be subject to epigenetic regulation for FE. Different from the results of previous RNA-seq-based studies [30,31,67,68], dual-transcription-related GO terms were identified in the co-expression (Figure 5 and Figure S4). Functional annotation and pathway analysis of these candidate genes provided crucial insights into the molecular processes influenced by DNA methylation concerning FE. Particularly, the key biological pathways related to nutrient uptake, metabolism, and cellular energy utilization emerged as potential targets for epigenetic regulation to improve FE. Integrating epigenetic and gene expression data allowed us to gain a more holistic view of the molecular bases of this economically significant trait, shedding light on the interplay between genetic and epigenetic factors.

Despite the valuable insights provided by our study, several intriguing avenues for further investigation remain. Given the complexity of FE, future research should consider expanding the integration of additional epigenetic marks and transcriptomic data from diverse tissues and developmental stages. The array used in the present study only focuses on genomic regions that are highly conserved among mammals. The highly divergent regions need to be analyzed in the future using a high-coverage array or whole-genome bisulfite sequencing [72]. Furthermore, the functional enrichment and co-expression results can only provide general indications of the roles of DNA methylation in RFI, and functional validation of the identified DMS and their impact on gene regulation through functional genomics approaches will be crucial in establishing causative relationships between DNA methylation and FE.

Our results mark a significant advancement in optimizing livestock production by harnessing the power of epigenetic regulation to enhance FE, reduce resource consumption, and promote global agricultural sustainability. Incorporating epigenetic insights into conventional genetic selection offers significant potential to propel the livestock industry toward increased efficiency and sustainability. This integration ensures a more promising future for food production and environmental stewardship.

## 5. Conclusions

Our study represents an initial assessment of the DNA methylome in cows with divergent RFI values, offering preliminary insights into the genetic basis of FE in cattle. Specific candidate genes and pathways associated with RFI identified here provide a potential roadmap for targeted breeding strategies to enhance FE and promote sustainable agricultural practices.

## Data Availability

Data is contained within the article or Supplementary Material.

## Supplementary Materials

The following supporting information can be downloaded at: https://www.mdpi.com/article/10.3390/genes14122121/s1, Figure S1: Heatmap of the expression of 286 FE genes across tissues; Figure S2: Determination of soft-threshold power in WGCNA; Figure S3: Gene number per module in the co-expression network; Figure S4: GO enrichment analysis for genes in the sub-co-expression network. Table S1: Detailed information about the methylation array; Table S2: 1730 CpG sites associated with residual feed intake using the categories approach; Table S3: 1636 CpG sites associated with residual feed intake using linear regression approach; Table S4: 421 overlapped CpG sites were identified using two approaches; Table S5: Correlation between gene expression and age at early and later stages; Table S6: The module of co-expression network; Table S7: GO enrichment of each module.
