# Supplementary material for: Unraveling the Genetic Basis of Feed Efficiency in Cattle through Integrated DNA Methylation and CattleGTEx Analysis
Source: Genes (Basel). 2023 Nov 24;14(12):2121. doi: 10.3390/genes14122121 (PMC10742843; doi:10.3390/genes14122121)
Supplement: Supplementary file 1 [file genes-14-02121-s001.zip › Supplimentary figure.pptx]

## Slide 1
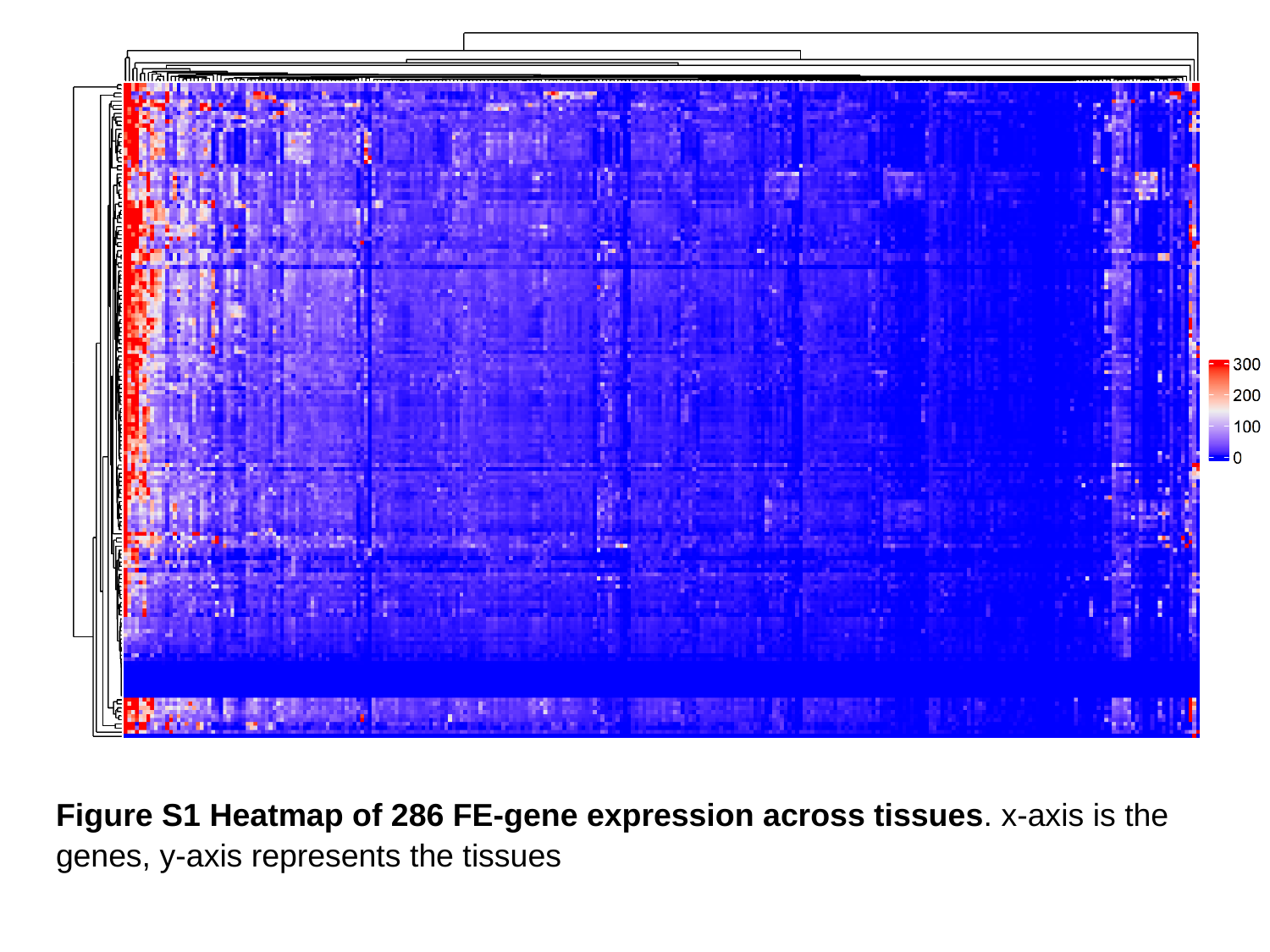

#
Figure S1 Heatmap of 286 FE-gene expression across tissues. x-axis is the genes, y-axis represents the tissues

## Slide 2
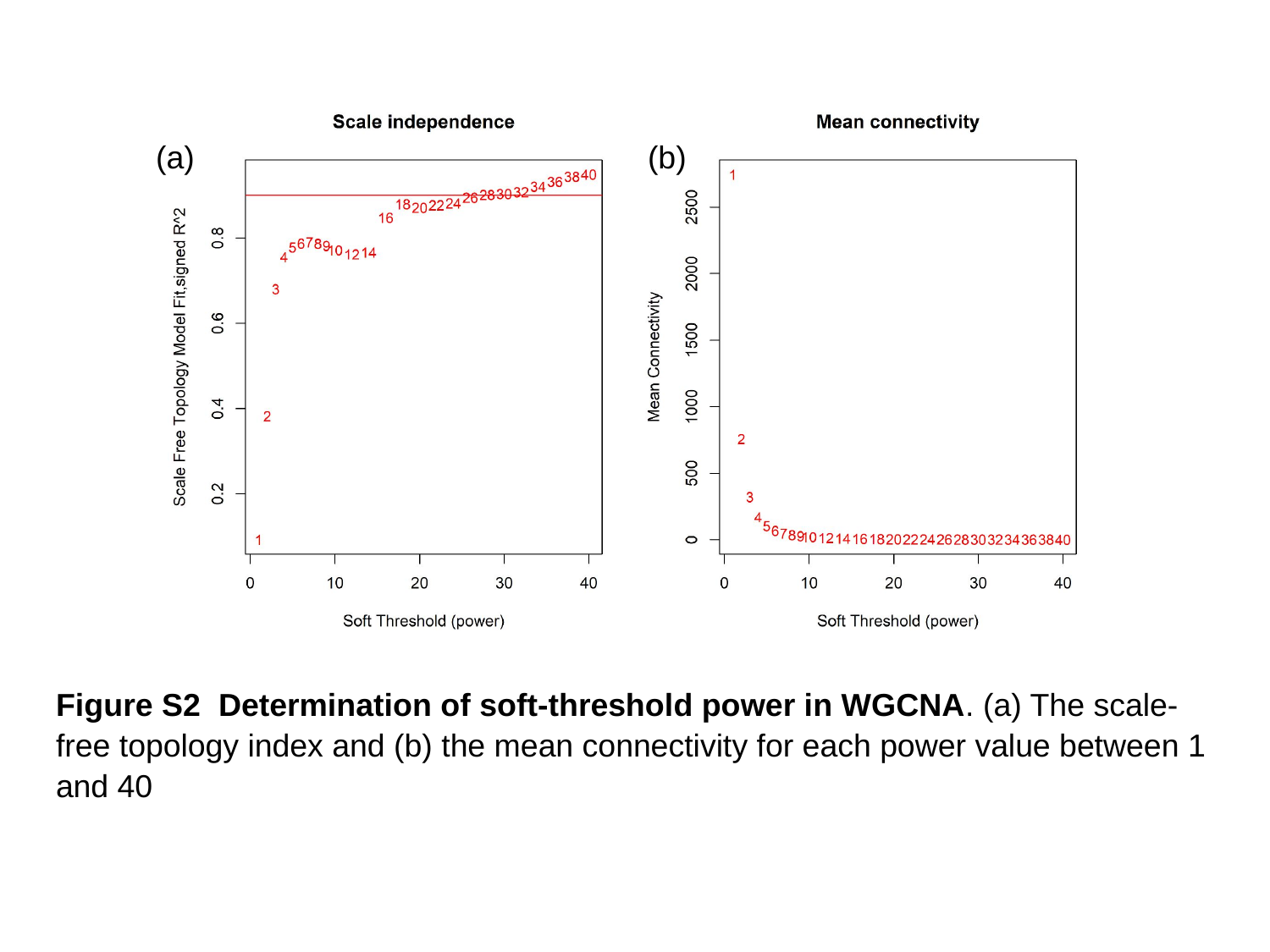

(a)
(b)
Figure S2 Determination of soft-threshold power in WGCNA. (a) The scale-free topology index and (b) the mean connectivity for each power value between 1 and 40

## Slide 3
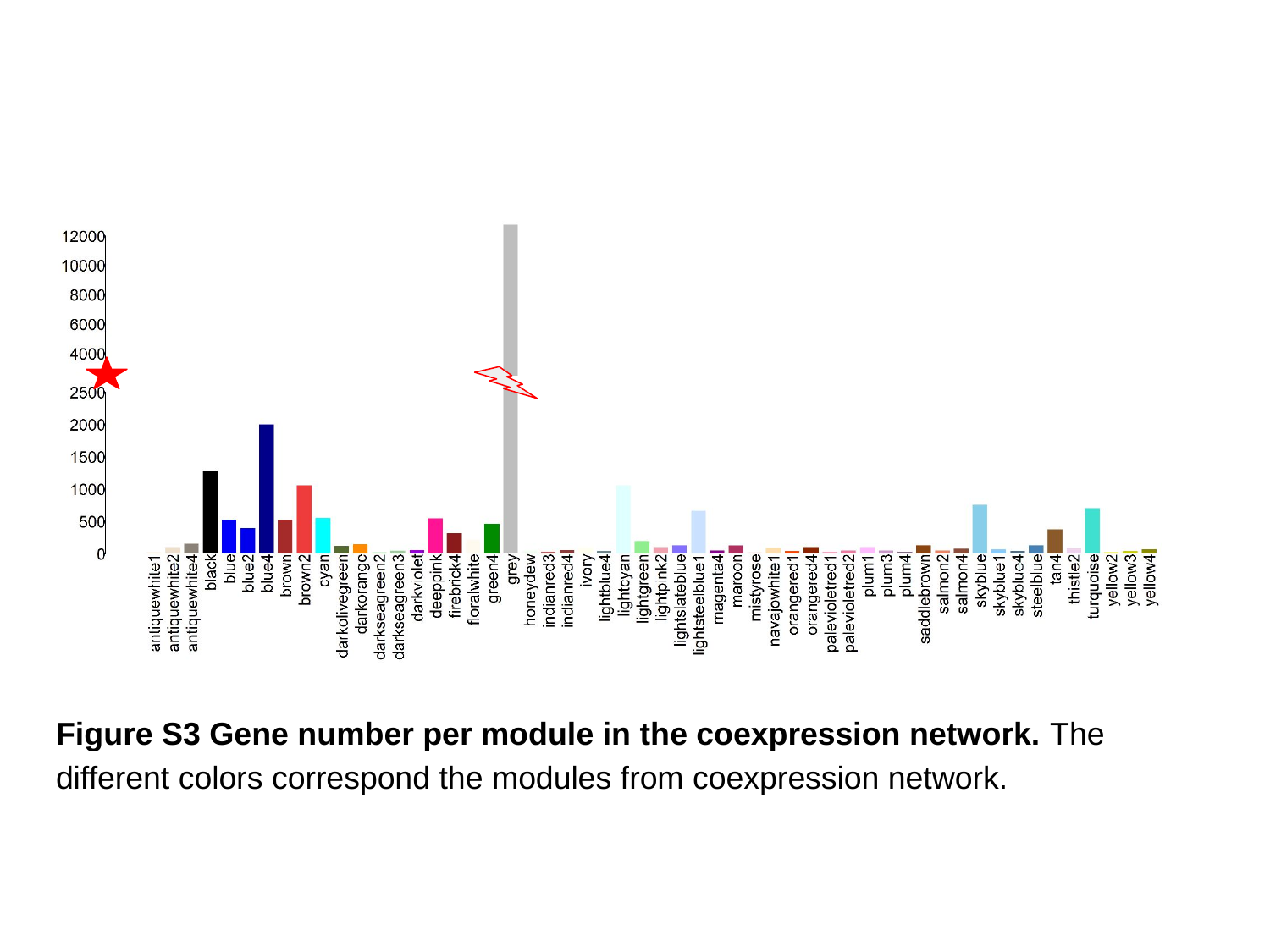

#
Figure S3 Gene number per module in the coexpression network. The different colors correspond the modules from coexpression network.

## Slide 4
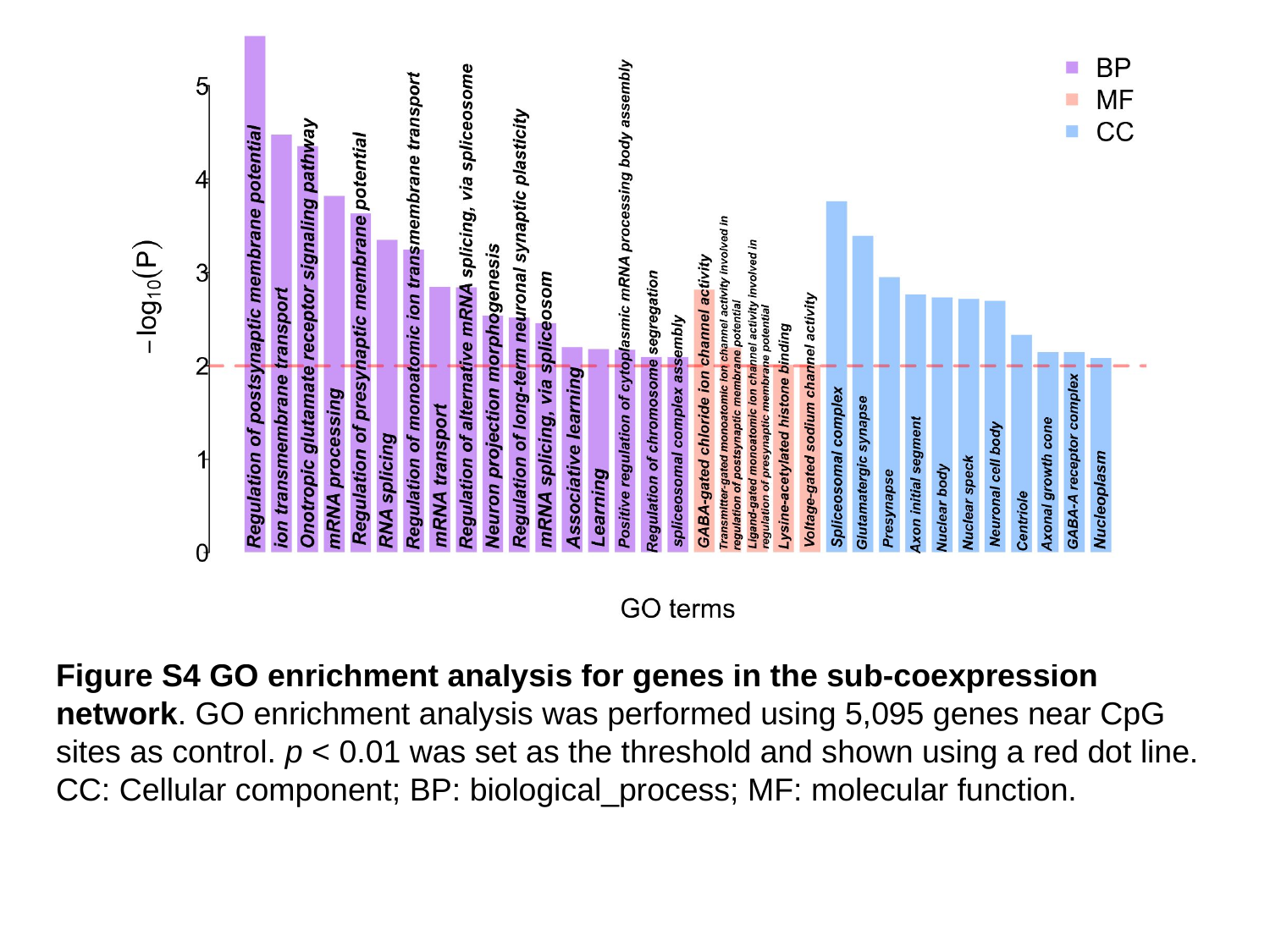

#
Figure S4 GO enrichment analysis for genes in the sub-coexpression network. GO enrichment analysis was performed using 5,095 genes near CpG sites as control. p < 0.01 was set as the threshold and shown using a red dot line. CC: Cellular component; BP: biological_process; MF: molecular function.
